# Progression of Protruding Plaque in Acute Coronary Syndrome Diagnosed by Serial Optical Coherence Tomography

**DOI:** 10.3390/jcm14217468

**Published:** 2025-10-22

**Authors:** Yuki Aoki, Norihito Nakamura, Sho Torii, Makoto Natsumeda, Frederic Turcotte-Gosselin, Manabu Shiozaki, Kaho Hashimoto, Daiki Suzuki, Ryosuke Omura, Kazuki Aihara, Katsuaki Sakai, Masataka Nakano, Gaku Nakazawa, Yuji Ikari

**Affiliations:** 1 Department of Cardiology, Tokai University School of Medicine, 143 Shimokasuya, Isehara 259-1193, Kanagawa, Japannatsumedaoverture1928@hotmail.com (M.N.); fred_t_g@hotmail.com (F.T.-G.);; 2 Department of Cardiology, Ikegami General Hospital, 6-1-19 Ikegami, Ota-ku 146-8531, Tokyo, Japan; 3 Nagasaki Heart Clinic, 4-1 Ebisumachi, Nagasaki 850-0056, Nagasaki, Japan; 4 Department of Cardiology, Ageo Chuo Medical Center, 1-10-10 Kashiwaza, Ageo 362-8588, Saitama, Japan; 5 Department of Cardiology, Faculty of Medicine, Kindai University, 377-2 Ohno-higashi, Osakasayama 589-8511, Osaka, Japan

**Keywords:** plaque protrusion, ACS, LDL, OCT, pro-atherosclerosis

## Abstract

**Background:** Plaque protrusion after stent implantation is frequently observed in acute coronary syndrome (ACS) patients, yet studies on its long-term progression and clinical significance are limited. **Methods:** Seventy-eight ACS patients underwent optical coherence tomography (OCT)-guided PCI and follow-up OCT at 1 year. A total of 101 protruding lesions were classified into atherogenic neointima (AN) and non-AN groups based on OCT findings. Qualitative and quantitative assessments of protruding plaque, including irregularity and plaque intensity, were conducted. **Results:** AN developed in 17% of irregular protrusion (IP) lesions, whereas no smooth protrusion progressed to AN. Lesions in the AN group showed greater increases in protruding plaque volume (2.80 ± 0.46 mm^2^ vs. 0.67 ± 0.16 mm^2^, *p* < 0.001) and diameter stenosis (16.5% vs. 10.1%, *p* = 0.02). Follow-up LDL levels were higher in the AN group compared with the non-AN group (76.9 vs. 61.2 mg/dL, *p* = 0.02), despite similar baseline levels. **Conclusions:** Low-intensity IP after stent implantation in ACS patients carries a high risk of progression to AN, particularly under poor LDL control. Aggressive lipid-lowering therapy may mitigate this risk.

## 1. Introduction

Percutaneous coronary intervention (PCI) with drug eluting stent (DES) implantation has been recommended as a first line therapy in patients with acute coronary syndrome (ACS) [1,2]. However, several studies have demonstrated that the risk of target lesion failure such as stent thrombosis (ST) and target lesion revascularization (TLR) after DES implantation was higher in patients with ACS compared with chronic coronary syndrome (CCS) [3].

Tissue protrusion (TP), defined as the extension of tissue into the lumen between stent struts upon intravascular imaging, has been identified as a significant factor contributing to both ST and TLR [4,5]. Observational studies suggest that TP is more prevalent in ACS patients due to higher plaque and thrombus volumes [6]. The incidence of TP has been reported to range from 17% to 70% when using intravascular ultrasound (IVUS) but this number rises to 90–95% when using optical coherence tomography (OCT) due to its higher resolution [5,7,8,9,10,11]. However, the clinical significance of TP evolving into atherosclerotic lesions, particularly in the context of serial OCT observations, remains poorly understood.

OCT provides higher axial resolution than IVUS, enabling precise detection of TP and detailed assessment of plaque morphology and neointimal patterns; therefore, OCT was chosen to evaluate baseline and 1-year changes in this ACS cohort.

To date, no studies have focused on serial imaging to investigate the prognosis of protruding plaque in ACS patients. Therefore, the purpose of this study is to evaluate the frequency and clinical outcomes of TP following stent implantation in ACS patients using baseline and one-year follow-up OCT.

### The Present Study Makes the Following Contributions

1. First serial OCT evaluation of protruding plaque in ACS: We provide the first systematic observation of TP following stent implantation in ACS patients, using serial OCT from baseline to one year.
2. Identification of pro-atherosclerosis progression: We demonstrate that low-intensity IP uniquely progresses to AN, introducing the novel concept of “pro-atherosclerosis” as a mechanistic pathway distinct from classical neoatherosclerosis.
3. Clinical implications for lipid management: We show that inadequate LDL control (>70 mg/dL) is strongly associated with this progression, highlighting the importance of intensive lipid-lowering strategies in ACS patients after PCI.

## 2. Methods

### 2.1. Study Design and Population

This was a single-center, observational cohort study that enrolled 127 consecutive patients with ACS who underwent OCT-guided PCI with newer-generation DES. Patients were selected based on their agreement to participate in a one-year follow-up evaluation, which involved the assesment of implanted stents using OCT. Exclusion criteria were as follows: (1) patient refusal, (2) patients with moderate to severe renal dysfunction, except for patients undergoing hemodialysis, and (3) poor OCT image quality. ACS was defined as ST-segment elevation myocardial infarction (STEMI), non-STEMI (NSTEMI), or unstable angina pectoris (UAP) based on previous guidelines [12]. PCI procedures were performed according to the current standard clinical guidelines [1,13] and the intervention strategy was at the discretion of the operator. Clinical characteristics, laboratory data, and medicinal information were collected from our institutional database. Finally, among those 78 patients who completed the 1-year follow-up OCT were selected, and a total of 101 protruding lesions assessed by initial OCT were divided into 2 groups: the AN group (*n* = 11) and the non-AN group (*n* = 90) (Figure S1).

### 2.2. PCI Procedure

All PCI procedures were performed according to current clinical guidelines and at the discretion of the operator. The type of DES used (durable polymer or biodegradable polymer) was selected based on clinical judgment. In cases where TP was observed after stent implantation, adjunctive therapies such as balloon post-dilatation were applied as needed.

### 2.3. OCT Imaging

OCT imaging was performed at baseline immediately following stent implantation and at the one-year follow-up using frequency-domain OCT systems (Dragonfly JP catheter and ILUMIEN OPTIS system, Abbott Vascular, Santa Clara, CA, USA; FastView catheter and LUNAWAVE system, Terumo, Tokyo, Japan). Imaging data were analyzed separately by two observers (Y.A. and N.N.) who were blinded to clinical and procedural information. Any discrepancies were resolved by consensus.

Key measurements included: minimum lumen area (MLA), stent area, protrusion area, and protrusion height. Qualitative and quantitative analyses were performed using standard criteria for OCT-based plaque characterization [14,15,16,17,18].

### 2.4. Evaluation of Intensity of Protrusion by ImageJ

The intensity of the protruding tissue evaluated with OCT after stent implantation for ACS was measured by using ImageJ software version 1.54 (National Institutes of Health, Bethesda, MD, USA). Color optic disc images were converted to gray scale images and overall disc brightness was measured, which converts gray scale images to intensity per pixel to calculate brightness values. The slice with the largest protrusion area by OCT was analyzed. A perpendicular line was drawn from the most protruding point to the stent strut for the measurement of the gray scale values (Figure S2). The protruding plaque was classified into high intensity and low intensity based on the mean gray scale value, with the cut-off value determined using receiver operating characteristic (ROC) curve. ROC curve analysis was performed to identify the cut-off value of the mean gray scale for predicting changes in AN. The optimal cut-off value was defined as the value that maximized Youden’s index.

### 2.5. Protrusion Classification

**Figure 1:**
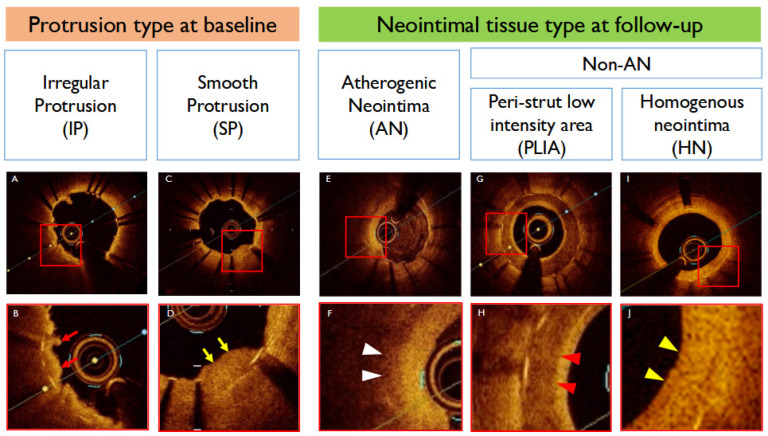
**Classifications of tissue protrusion and neointimal tissue types in OCT.** Irregular protrusion (IP) was characterized by material with an irregular surface extruding into the lumen between the stent struts (**A**,**B**) (red arrow), whereas SP was identified by extruding plaque with a smooth surface (**C**,**D**) (yellow arrow). Neointimal tissue at the one-year follow-up was classified into three patterns: atherogenic neointima (AN), homogeneous neointima (HN), and PLIA (peri-strut low-intensity area). AN was characterized by a signal-poor region with diffuse border and invisible stent struts underneath (**E**,**F**) (white arrowhead). PLIA demonstrated regions around the stent struts with a homogeneous, low-intensity signal compared to surrounding tissue without signal attenuation (**G**,**H**) (red arrowhead). HN showed homogeneous neointimal patterns with uniform optical properties (**I**,**J**) (yellow arrowhead). Red squares indicate the areas of interest corresponding to the detailed images shown in adjacent panels. OCT: optical coherence tomography; IP: irregular protrusion; AN: atherogenic neointima; HN: homogeneous neointima; PLIA: peri-strut low-intensity area.

TP is commonly defined as tissue extension into the lumen through stent struts after stent implantation. Protruding lesions were further classified based on their morphology as IP or Smooth protrusion (SP). IP was defined as protrusion of material with an irregular surface into the lumen between stent struts, whereas SP was identified by protruding plaque with a smooth surface [4] (Figure 1). Additionally, lesions were divided into two categories based on intensity: low-intensity and high-intensity, using ImageJ software (NIH, Bethesda, MD, USA). The intensity cutoff for low- and high-intensity protrusions was determined using receiver operating characteristic (ROC) analysis.

At the one-year follow-up, neointimal tissue was categorized into three patterns: AN, peri-strut low-intensity area (PLIA), and homogeneous neointima (HN) as per previously validated criteria [19,20] (Figure 1). In brief, AN was defined as a signal-poor lesion with diffuse border and invisible stent struts underneath. PLIA was defined as regions around the stent struts with a homogeneous, low-intensity signal compared to surrounding tissue without signal attenuation. HN was defined as homogeneous neointimal patterns with uniform optical properties without focal variation in backscattering pattern.

### 2.6. Quantitative Coronary Angiography (QCA)

QCA analysis was performed at baseline and the one-year follow-up using Medis CMS software version 7.0 (Medis Medical Imaging Systems, Leiden, The Netherlands). Lesion characteristics such as lesion length, reference diameter, minimum lumen diameter, and diameter stenosis were measured. Acute gain and late lumen loss were calculated. QCA of both the baseline and 1-year follow up were independently performed by two experienced cardiologists.

### 2.7. Statistical Analysis

Continuous variables were expressed as mean ± standard deviation (SD) or median (interquartile range) depending on their distribution, which was assessed using the Shapiro–Wilk test. Categorical variables were expressed as frequencies and percentages. Comparisons between groups were performed using Student’s *t*-test for normally distributed continuous variables and the Mann–Whitney U test for non-normally distributed variables. Categorical variables were compared using the chi-square test or Fisher’s exact test as appropriate.

Intra- and inter-observer agreement for OCT measurements were assessed using Cohen’s kappa coefficient (κ). A kappa value of 0.61–0.80 indicated good agreement, and values above 0.81 indicated excellent agreement. Statistical significance was set at *p* < 0.05. Analyses were conducted using JMP software version 16.0 (SAS Institute, Cary, NC, USA) and GraphPad Prism software version 7.03 (GraphPad Software, San Diego, CA, USA).

### 2.8. Ethical Approval

The study was conducted in accordance with the Declaration of Helsinki and approved by the institutional review board of Tokai University (IRB identifier: 16R-032). Written informed consent was obtained from all patients prior to participation.

## 3. Results

### 3.1. Baseline and Procedural Characteristics

A total of 78 ACS patients with 101 lesions were included. The majority presented with STEMI (80.2%), followed by NSTEMI (11.9%) and UAP (7.9%). Baseline patient characteristics are summarized in Table 1, and lesion-level characteristics with QCA are presented in Table 2. There were no significant differences in age, sex, clinical presentation, or cardiovascular risk factors between the AN and non-AN groups. Baseline lipid levels, including LDL-C, HDL-C, and triglycerides, were similar between the two groups. Among the 78 patients, 24 (30.8%) contributed more than one lesion (23 with two; 1 with three). At the patient level, any AN at 1 year occurred in 11/78 (14.1%), and no patient had more than one AN lesion. No periprocedural complications—flow-limiting dissection, perforation, or no-reflow requiring therapy—occurred during the index PCI, making confounding of serial OCT findings or subsequent outcomes unlikely. Procedural characteristics, including stent platforms, balloon sizes and pressures, and rates of pre-/post-dilatation, were comparable between the AN and non-AN groups.

### 3.2. OCT Findings at Baseline and 1-Year Follow-Up

#### 3.2.1. Baseline OCT Findings

TP was detected in all 101 lesions at baseline, with 64% (65 lesions) classified as IP. Baseline quantitative OCT findings are shown in Table 3. Protrusion height was significantly greater in the AN group compared to the non-AN group (0.52 ± 0.05 mm vs. 0.39 ± 0.01 mm, *p* = 0.01), although protrusion length and area were similar between the two groups. All other baseline characteristics, including reference lumen area, stent size, and stent diameter, were comparable.

#### 3.2.2. Follow-Up OCT Findings

**Figure 2:**
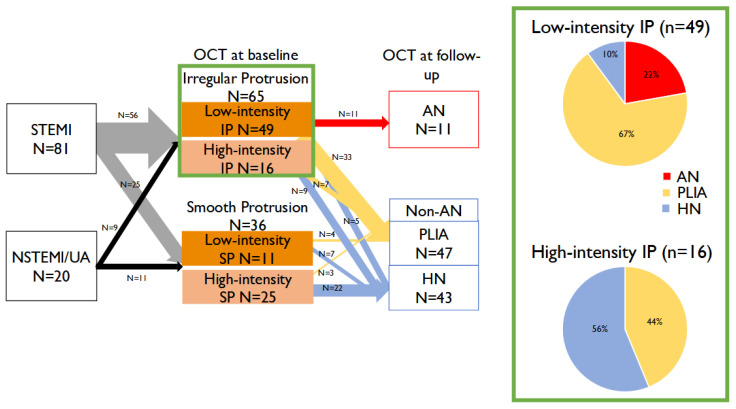
**Qualitative changes in protruding plaque after 1 year.** Among ST-segment elevation myocardial infarction lesions, irregular protrusion (IP) was observed in 56 lesions (69%), while a lower incidence of IP was found in non-ST-segment elevation myocardial infarction/unstable angina lesions (45%). A higher incidence of low-intensity IP was seen in IP compared to SP (75% versus 31%, respectively). Additionally, 17% of IP lesions had progressed to atherogenic neointima (AN), whereas no SP lesion progressed to AN at follow-up. IP: irregular protrusion; AN: atherogenic neointima; HIP: high-intensity irregular protrusion; HN: homogenous neointima; LIP: low-intensity irregular protrusion; NSTEMI: non-ST-segment elevation myocardial infarction; OCT: optical coherence tomography; PLIA: peri-strut low-intensity area; STEMI: ST-segment elevation myocardial infarction; UA: unstable angina.

**Figure 3:**
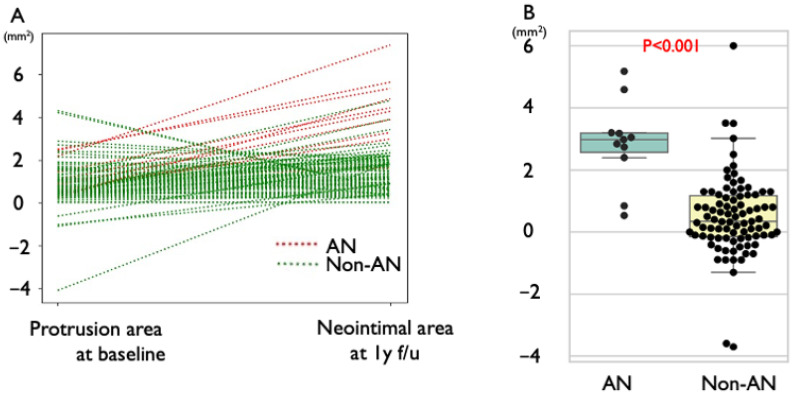
**Progression from protruding plaque to atherogenic neointima after stent implantation in patients with acute coronary syndrome.** The protrusion area was measured at the onset of acute coronary syndrome (ACS), and the neointimal area was measured at the 1-year follow-up. The green line represents the change in atherogenic neointima (AN), while the red line indicates the change in non-AN (**A**). The change in plaque volume was significantly greater in AN compared to non-AN (**B**). ACS: acute coronary syndrome; AN: atherogenic neointima.

The quantitative analysis of OCT at 1-year follow-up is shown in Table 3. Among STEMI lesions, IP was observed in 56 lesions (69%), while a lower incidence of IP was found in NSTEMI/UA lesions (45%). A higher incidence of low-intensity IP was seen in IP compared to SP (75% versus 31%, respectively). Additionally, 17% of IP lesions had progressed to AN, whereas no SP lesion progressed to AN at follow-up. Qualitative OCT changes are demonstrated in Figure 2. The increase in protruding plaque volume relative to the neointimal area was significantly greater in the AN group compared to the non-AN group (2.80 ± 0.46 mm^2^ vs. 0.67 ± 0.16 mm^2^, *p* < 0.001), resulting in significantly higher diameter stenosis (16.5 ± 0.87% vs. 10.1 ± 2.62%, *p* = 0.02). In all cases of the AN group, the area of signal-poor lesion with diffuse borders in the neointima at follow-up exceeded the protrusion area at baseline, indicating progression of the atherosclerotic lesion (Figure 3).

#### 3.2.3. Quantitative Coronary Angiography (QCA) Findings

QCA analysis revealed no significant differences in baseline metrics, including reference diameter, minimal lumen diameter (MLD), and diameter stenosis, between the AN and non-AN groups (Table 2). However, the AN group showed significantly greater acute gain after stent implantation compared to the non-AN group (1.92 ± 0.18 mm vs. 1.53 ± 0.06 mm, *p* = 0.01).

At the 1-year follow-up, the AN group had significantly higher late lumen loss compared to the non-AN group (0.76 ± 0.14 mm vs. −0.01 ± 0.05 mm, *p* = 0.01). Diameter stenosis at follow-up was also numerically higher in the AN group (37.4 ± 3.72% vs. 17.4 ± 1.30%, *p* = 0.06).

### 3.3. Gray Value Analysis of Protruding Plaque

Gray value analysis using ImageJ showed that the mean gray value in the AN group was significantly lower than in the non-AN group (108.8 ± 7.14 vs. 125.1 ± 2.61, *p* = 0.046). ROC curve analysis identified an optimal gray value cutoff of 87.2 for predicting the development of AN (sensitivity 100%, specificity 46.1%, AUC = 0.76, *p* < 0.01) (Supplemental Figure S3). All lesions that progressed to AN originated from low-intensity IP.

### 3.4. Clinical Outcomes

No major adverse cardiac events (MACE) occurred during the one-year follow-up period in either group, including cardiac death, non-fatal myocardial infarction, target lesion revascularization (TLR), or stent thrombosis. Medications at one year were similar between the groups, with no significant differences in the use of statins, antiplatelet therapy, or ezetimibe (Table 1).

### 3.5. Lipid Profile and Neointimal Progression

Although baseline LDL cholesterol levels were comparable between the AN and non-AN groups, follow-up LDL levels were significantly higher in the AN group (76.9 ± 4.80 mg/dL vs. 61.2 ± 1.70 mg/dL, *p* = 0.02) (Table 1, Supplemental Figure S4). Total cholesterol and non-HDL cholesterol were also higher in the AN group at one year, suggesting the importance of LDL control in preventing neointimal progression.

## 4. Discussion

This study provides serial observation of TP in ACS patients using OCT. We found that IP were present in 64% of cases following stent implantation and 17% of these IP lesions progressed to AN after one year. Importantly, all instances of progression originated from low-intensity IP, and patients with poor LDL control were significantly more likely to experience atherosclerotic plaque progression at 1-year follow up.

Our findings are consistent with prior studies that have identified IP as a significant risk factor for TLR and ST in patients with ACS [4,5]. Previous IVUS studies reported TP incidence rates ranging from 17–70% [7,8,9]. However, with the higher resolution of OCT, TP was observed in 90–95% of cases [5,10,11], similar to the current study. The reported prevalence of IP as a risk factor for TLR varies from 2–80% [4,17,18], and our study aligns with this range with a prevalence of 64.3%. These findings may be influenced by differences in patient backgrounds, as ACS lesions tend to be more vulnerable due to the greater prevalence of lipid-rich plaques and thrombi, contributing to an increased risk of TP. Specifically, prior studies have shown that post-stent IP and smaller minimum stent area independently predict 1-year device-oriented endpoints, that post-stent IP is associated with subsequent neoatherosclerosis on serial OCT at 8 months, and that early IP relates to later neointimal characteristics on follow-up OCT [4,5,17,20]. In an ACS-specific context, prior studies have documented serial changes in TP on follow-up OCT and evaluated serial IP change with its longer-term clinical impact [21,22]. Accordingly, rather than asserting primacy, we view our ACS-only cohort as complementary to prior serial OCT work by coupling a simple intensity-based stratification with a 1-year neointimal endpoint.

**Figure 4:**
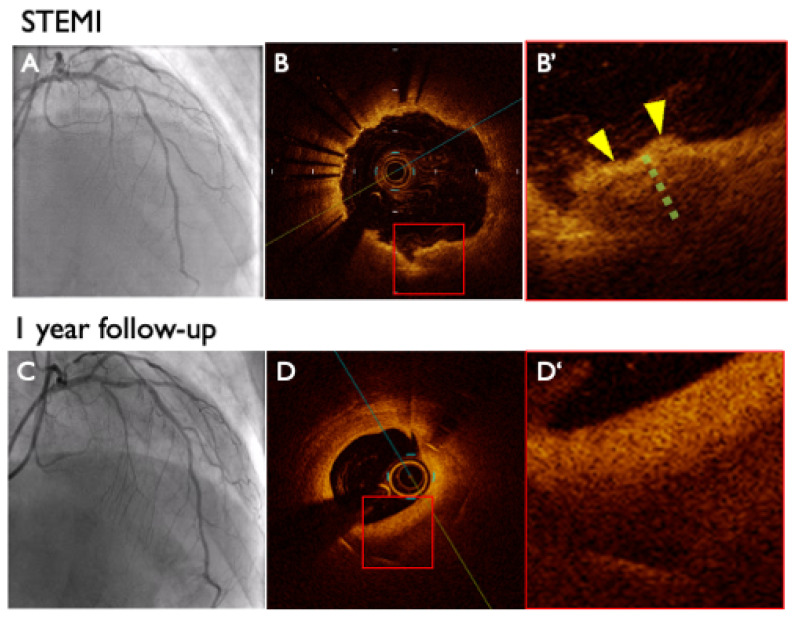
**Representative case of atherogenic neointima.** A 77-year-old man underwent primary percutaneous coronary intervention for ST-segment elevation myocardial infarction (**A**), which resulted in the development of irregular protrusion (IP) (**B**). OCT showed a low-intensity IP at the most protruding plaque (yellow arrowhead) with a mean gray value of 64.9 (green line) (**B’**). At follow-up (**C**), OCT revealed the presence of atherogenic neointima with increased plaque volume (**D**,**D’**). STEMI: ST-segment elevation myocardial infarction; IP: irregular protrusion; OCT: optical coherence tomography.

**Figure 5:**
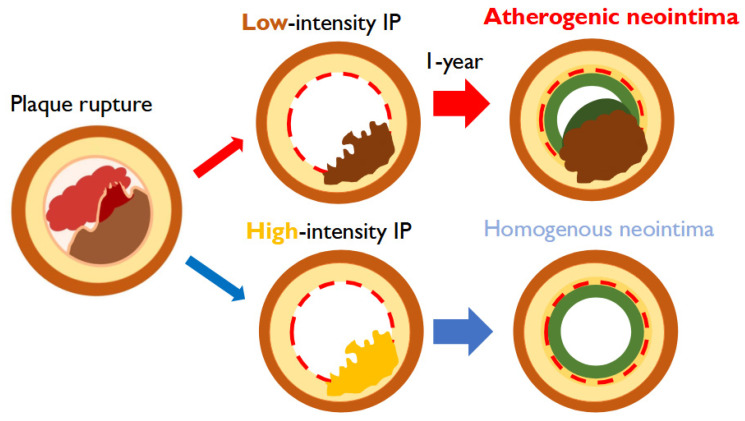
**Progression from protruding plaque to atherogenic neointima.** Low-intensity irregular protrusion (IP) following stent implantation at acute coronary syndrome can progress to atherogenic neointima (AN) with increased plaque volume. Conversely, high-intensity IP may lead to non-AN. IP: irregular protrusion; AN: atherogenic neointima.

The transition from IP to AN is an important mechanistic finding in this study. Pathologically, neoatherosclerosis has been described as the development of “new” lipid-laden neointima following stent implantation [19,20,21]. However, rather than a different phenomenon, the OCT appearance we labeled as AN substantially overlaps with OCT-defined neoatherosclerosis. Accordingly, we use ‘pro-atherosclerosis’ not as a new pathological entity but as a conceptual, serial-imaging framework that describes the morphologic evolution from low-intensity IP toward a lipid-laden neointimal phenotype over time (Figure 4 and Figure 5). This progression was observed exclusively in low-intensity IP lesions and was associated with elevated LDL levels at the one-year follow-up, suggesting that lipid infiltration may play a key role in advancing plaque progression within these lesions. These findings suggest that serial OCT may have value for detecting this progression in clinical practice, but prospective validation is needed.

Our findings suggest that poor LDL control (>70 mg/dL) during follow-up is a crucial factor in the transition from IP to AN. This mechanism may involve impaired endothelial function and subsequent lipoprotein infiltration, which has previously been implicated in neoatherosclerosis formation [20,22]. Given that low-intensity IP lesions are at higher risk for developing into AN, it is crucial to implement aggressive lipid-lowering strategies in ACS patients post-stent implantation, especially for those exhibiting TP on OCT imaging. Further research should explore whether earlier intervention with potent lipid-lowering agents, such as PCSK9 inhibitors, might reduce the risk of AN development in patients with IP lesions. Although our study did not include patients treated with these newer agents, future trials could evaluate their efficacy in slowing plaque progression and improving long-term outcomes.

## 5. Limitation

This study has several limitations. First, it was conducted at a single center, which may limit generalizability. Second, although OCT provides high-resolution intravascular imaging, the interpretation of TP immediately after stent implantation can be confounded by residual thrombus, potentially overestimating TP prevalence. Third, only 78 of 127 enrolled patients completed the 1-year OCT; therefore, attrition may have introduced selection bias (reasons summarized in Supplementary Figure S1). Fourth, analyses were primarily lesion-level and the number of AN lesions was small (*n* = 11); thus, multivariable or mixed-effects modeling was not performed to avoid overfitting, within-patient clustering may have led to imprecise variance estimates, and the findings should be considered hypothesis-generating. Fifth, two OCT systems were used; because absolute gray-value measurements can vary by device and gain, the ROC-derived intensity cutoff (87.2) is cohort-specific and may not generalize without harmonization or external validation. Sixth, operator discretion (e.g., post-dilatation, thrombus aspiration) and heterogeneous stent platforms could have influenced protrusion morphology and healing, and multiple unadjusted comparisons and unmeasured confounding may remain. Finally, the study was not event-powered and no 1-year MACE occurred, so the clinical implications are inferential. Larger, multicenter, event-powered studies with standardized intensity calibration are warranted.

## 6. Conclusions

In conclusion, this study provides novel insights into the prognosis of TP following stent implantation in ACS patients. Our findings suggest that low-intensity IP lesions are especially susceptible to progress to AN. Poor LDL control appears to be a significant factor in this progression, highlighting the importance of intensive lipid-lowering strategies in high-risk ACS patients.

## Figures and Tables

**Table 1:** Baseline patient characteristics and 1-year medications/lipid profiles.

	Overall	AN (*n* = 11)	Non-AN (*n* = 90)	*p*
Age, mean ± SD, y	63.6 ± 11.5	67.8 ± 3.42	63.1 ± 1.21	0.14
Male sex, *n* (%)	83.0 (82.2)	8.0 (72.7)	75.0 (83.3)	0.41
BMI, mean ± SD, kg/m^2^	24.6 ± 3.01	25.5 ± 0.89	24.4 ± 0.32	0.17
Clinical presentation, *n* (%)				
STEMI, *n* (%)	81.0 (80.2)	8.0 (72.7)	73.0 (81.1)	0.51
NSTE-ACS, *n* (%)	20.0 (19.8)	3.0 (27.3)	17.0 (18.9)	0.45
Previous PCI, *n* (%)	1.0 (0.99)	0.0 (0.0)	1.0 (1.1)	1.0
Previous CABG, *n* (%)	0.0 (0.0)	0.0 (0.0)	0.0 (0.0)	NA
Hypertension, *n* (%)	65.0 (64.4)	8.0 (72.7)	57.0 (63.3)	0.60
Dyslipidemia, *n* (%)	75.0 (74.3)	11.0 (100.0)	64.0 (71.1)	0.01
Diabetes mellitus, *n* (%)	21.0 (20.8)	6.0 (54.5)	15.0 (16.6)	0.01
Chronic kidney disease, *n* (%)	25.0 (24.8)	3.0 (27.3)	22.0 (24.4)	0.87
Dialysis, *n* (%)	0.0 (0.0)	0.0 (0.0)	0.0 (0.0)	NA
Smoking history, *n* (%)	63.0 (62.4)	5.0 (45.5)	58.0 (64.4)	0.18
LVEF, mean ± SD, %	63.3 ± 10.1	62.8 ± 10.0	65.2 ± 9.79	0.27
Laboratory data at admission				
Hb, g/dL	14.5 ± 1.82	14.6 ± 0.50	14.4 ± 0.24	0.93
Cre, mg/dL	0.92 ± 0.25	0.82 ± 0.11	0.87± 0.02	0.44
Total cholesterol, mg/dL	203.1 ± 34.4	193.0 ± 10.3	205.5 ± 4.60	0.28
HDL cholesterol, mg/dL	51.1 ± 12.1	50.6 ± 3.72	51.1 ± 1.30	0.84
Non-HDL cholesterol, mg/dL	153.1 ± 35.0	142.4 ± 10.5	155.6 ± 4.72	0.18
LDL cholesterol, mg/dL	127.0 ± 32.4	117.6 ± 9.81	128.3 ± 3.50	0.22
Triglyceride, mg/dL	151.8 ± 15.5	136.7 ± 48.1	154.7 ± 16.4	0.43
HbA1c, %	6.3 ± 1.42	6.6 ± 1.63	6.8 ± 0.55	0.14
Medications at 1-year follow-up				
Aspirin, *n* (%)	69.0 (69.7)	7.0 (63.6)	62.0 (68.8)	0.65
P2Y12, *n* (%)	82.0 (82.8)	10.0 (90.9)	72.0 (80.0)	0.45
Anticoagulant, *n* (%)	6.0 (1.7)	0.0 (0.0)	6.0 (6.66)	0.37
RAS inhibitor, *n* (%)	96.0 (95.0)	11.0 (100.0)	85.0 (94.4)	0.53
Beta blocker, *n* (%)	91.0 (90.1)	11.0 (100.0)	80.0 (88.9)	0.29
Statin, *n* (%)	101.0 (100)	11.0 (100.0)	90.0 (100.0)	1.0
Ezetimibe, *n* (%)	35.0 (34.7)	2.0 (18.2)	32.0 (35.6)	0.54
PCSK9 inhibitor, *n* (%)	0.0	0.0 (0.0)	0.0 (0.0)	NA
Laboratory data at 1-year follow-up				
Total cholesterol, mg/dL	140.3 ± 25.9	162.5 ± 7.92	136.3 ± 2.91	0.01
HDL cholesterol, mg/dL	56.2 ± 12.1	54.5 ± 3.71	56.4 ± 1.30	0.65
Non-HDL cholesterol, mg/dL	99.3 ± 30.3	114.3 ± 8.02	85.8 ± 2.90	0.01
LDL cholesterol, mg/dL	63.7 ± 18.7	76.9 ± 4.80	61.2 ± 1.70	0.02
Triglyceride, mg/dL	139.7 ± 74.4	156.7 ± 22.5	137.8 ± 7.91	0.26
HbA1c, %	6.1 ± 0.63	6.3 ± 0.19	6.1 ± 0.06	0.22

Values are expressed as mean ± standard deviation or *n* (%). BMI = body mass index; STEMI = ST-segment elevation myocardial infarction; NSTE-ACS = non-ST-segment elevation- acute coronary syndrome; PCI = percutaneous coronary intervention; CABG = Coronary artery bypass grafting; LVEF = left ventricular ejection fraction; HDL = high-density lipoprotein; LDL = low-density lipoprotein; RAS = renin angiotensin system; PCSK-9 = proprotein convertase subtilisin kexin type 9.

**Table 2:** Baseline lesion characteristics and QCA.

	Overall	AN (*n* = 11)	Non-AN (*n* = 90)	*p*
Target vessel				0.11
LMCA, *n* (%)	1.0 (0.99)	0.0 (0.0)	1.0 (1.11)	
LAD, *n* (%)	68.0 (67.3)	5.0 (45.5)	63.0 (70.0)	
LCX, *n* (%)	7.0 (6.93)	0.0 (0.0)	7.0 (7.77)	
RCA, *n* (%)	25.0 (24.8)	6.0 (54.5)	19.0 (21.1)	
Stent				0.46
DP-DES, *n* (%)	37.0 (36.6)	5.0 (45.5)	32.0 (35.6)	
BP-DES, *n* (%)	64.0 (63.4)	6.0 (54.5)	58.0 (64.4)	
QCA				
Baseline				
Reference proximal diameter (mm)	2.9 ± 0.63	2.6 ± 0.19	2.9 ± 0.06	0.36
Reference distal diameter (mm)	2.4 ± 0.51	2.3 ± 0.16	2.4 ± 0.05	0.38
Minimal lumen diameter (mm)	0.98 ± 0.36	0.79 ± 0.11	1.0 ± 0.03	0.07
Diameter stenosis (%)	59.8 ± 15.7	64.2 ± 4.92	59.3 ± 1.60	0.41
After procedure				
Stent proximal diameter (mm)	3.1 ± 0.48	3.2 ± 0.15	3.1 ± 0.05	0.39
Stent distal diameter (mm)	2.8 ± 0.47	2.9 ± 0.15	2.8 ± 0.05	0.16
Minimal lumen diameter (mm)	2.5 ± 0.44	2.7 ± 0.13	2.5 ± 0.04	0.03
Diameter stenosis (%)	15.9 ± 8.31	10.1 ± 2.62	16.5 ± 0.87	0.02
Acute gain (mm)	1.54 ± 0.60	1.92 ± 0.18	1.53 ± 0.06	0.01
At 1-year follow-up				
Stent proximal diameter (mm)	3.1 ± 0.52	3.1 ± 0.16	3.1 ± 0.05	0.72
Stent distal diameter (mm)	2.8 ± 0.54	2.8 ± 0.17	2.8 ± 0.05	0.74
Minimal lumen diameter (mm)	2.4 ± 0.63	1.9 ± 0.19	2.5 ± 0.06	0.13
Diameter stenosis (%)	19.4 ± 13.3	37.4 ± 3.72	17.4 ± 1.30	0.06
Late loss (mm)	0.08 ± 0.52	0.76 ± 0.14	−0.01 ± 0.05	0.01

LMCA = left main coronary artery; LAD = left anterior descending coronary artery; LCX = left circumflex coronary artery; RCA = right coronary artery; DP-DES = durable polymer drug-eluting stent; BP-DES = Bioresorbable Polymer Drug-Eluting Stent; QCA = Quantitative Coronary Angiography.

**Table 3:** OCT Findings.

	Overall	AN (*n* = 11)	Non-AN (*n* = 90)	*p*
Pre-PCI				
Etiology				0.28
Plaque rupture, *n* (%)	80.0 (79.2)	11.0 (100.0)	69.0 (76.6)	
Erosion, *n* (%)	3.0 (2.97)	0.0 (0.0)	3.0 (3.33)	
Calcified nodule, *n* (%)	0.0 (0.0)	0.0 (0.0)	0.0 (0.0)	
Others, *n* (%)	18.0 (17.8)	0.0 (0.0)	18.0 (20.0)	
Plaque morphologies				0.43
Lipid rich Plaque, *n* (%)	83.0 (82.2)	11.0 (100.0)	72.0 (80.0)	
Fibrous Plaque, *n* (%)	9.0 (8.91)	0.0 (0.0)	9.0 (10.0)	
Calcification, *n* (%)	2.0 (1.98)	0.0 (0.0)	2.0 (2.22)	
Others, *n* (%)	7.0 (6.93)	0.0 (0.0)	7.0 (7.77)	
Angiographic findings				
Pre TIMI flow grade 0/1 *n* (%)	66.0 (65.3)	3.0 (27.2)	63.0 (70.0)	0.01
Final TIMI flow grade 3 *n* (%)	95.0 (94.1)	11.0 (100.0)	84.0 (93.3)	0.67
Lesion length, mm	25.0 ± 9.96	20.0 ± 2.92	25.6 ± 1.13	0.03
Proximal reference lumen area, mm^2^	14.0 ± 6.20	13.9 ± 37.8	28.1 ± 13.4	0.70
Distal reference lumen area, mm^2^	10.0 ± 4.92	11.5 ± 14.5	15.3 ± 5.10	0.35
Minimum lumen area, mm^2^	3.27 ± 2.50	3.46 ± 0.74	3.34 ± 0.27	0.52
Procedural details				
Thrombus aspiration, *n* (%)	63.0 (62.4)	5.0 (45.5)	58.0 (64.4)	0.22
Excimer laser coronary angioplasty (ELCA), *n* (%)	19.0 (18.8)	0.0 (0.0)	19.0 (21.1)	0.09
Balloon				
Pre-POBA, *n* (%)	75.0 (74.3)	9.0 (81.8)	66.0 (73.3)	0.54
Balloon pressure, atm	11.5 ± 0.62	10.6 ± 1.3	11.1 ± 0.42	0.64
Balloon size, mm	2.41 ± 0.37	2.51 ± 0.17	2.30 ± 0.05	0.44
Balloon length, mm	14.8 ± 0.72	15.0 ± 0.55	14.9 ± 0.18	0.74
Post POBA, *n* (%)	65.0 (64.4)	5.0 (45.5)	60.0 (66.6)	0.17
Balloon pressure	17.9 ± 0.82	15.5 ± 2.3	18.1 ± 0.75	0.28
Balloon size, mm	3.42 ± 0.72	3.41 ± 0.36	3.42 ± 0.11	0.74
Balloon length, mm	11.4± 0.75	10.7± 1.8	11.2 ± 0.62	0.85
Post-PCI				
Quantitative analysis				
Lumen area at most protruding site, mm^2^	6.94 ± 2.3	7.87 ± 0.67	6.84 ± 0.24	0.28
Stent area at most protruding site, mm^2^	7.76 ± 2.6	9.19 ± 0.76	7.74 ± 0.27	0.19
Stent length, mm	27.2 ± 12.3	23.5 ± 3.1	27.7 ± 1.1	0.19
Stent diameter, mm	3.12 ± 0.44	3.30 ± 0.13	3.14 ± 0.05	0.20
Protrusion analysis				
Protrusion height mm	0.41 ± 0.20	0.52 ± 0.05	0.39 ± 0.01	0.01
Protrusion length mm	3.10 ± 2.8	2.54 ± 0.99	3.50 ± 0.34	0.75
Most Protrusion area, mm^2^	75.0 ± 65.3	80.8 ± 22.9	78.7 ± 8.1	0.19
1-year follow-up				
Minimal lumen area, mm^2^	5.87 ± 2.18	5.14 ± 0.67	6.14 ± 0.24	0.18
Neointimal area, mm^2^	1.63 ± 1.30	2.80 ± 0.46	0.67 ± 0.16	<0.001
Stent area, mm^2^	7.72 ± 2.50	9.21 ± 0.73	7.40 ± 0.26	0.06
% area stenosis, %	22.2 ± 14.6	46.6 ± 3.62	19.1 ± 1.31	<0.001

OCT = optical coherence tomography; PCI = percutaneous coronary intervention; TIMI = Thrombolysis in Myocardial Infarction; POBA = Plain Old Balloon Angioplasty.

## Data Availability

The data underlying this article will be shared on reasonable request to the corresponding author.

## Supplementary Materials

The following supporting information can be downloaded at: https://www.mdpi.com/article/10.3390/jcm14217468/s1.

## Abbreviations

ACS	acute coronary syndrome
AN	atherogenic neointima
HDL-C	high-density lipoprotein cholesterol
IP	irregular protrusion
LDL-C	low-density lipoprotein cholesterol
OCT	optical coherence tomography
PCI	percutaneous coronary intervention
TP	tissue protrusion
