# Supplementary material for: Progression of Protruding Plaque in Acute Coronary Syndrome Diagnosed by Serial Optical Coherence Tomography
Source: J Clin Med. 2025 Oct 22;14(21):7468. doi: 10.3390/jcm14217468 (PMC12608650; doi:10.3390/jcm14217468)
Supplement: Supplementary file 1 [file jcm-14-07468-s001.zip › jcm-3863460-supplementary.pdf]

Figure S-1

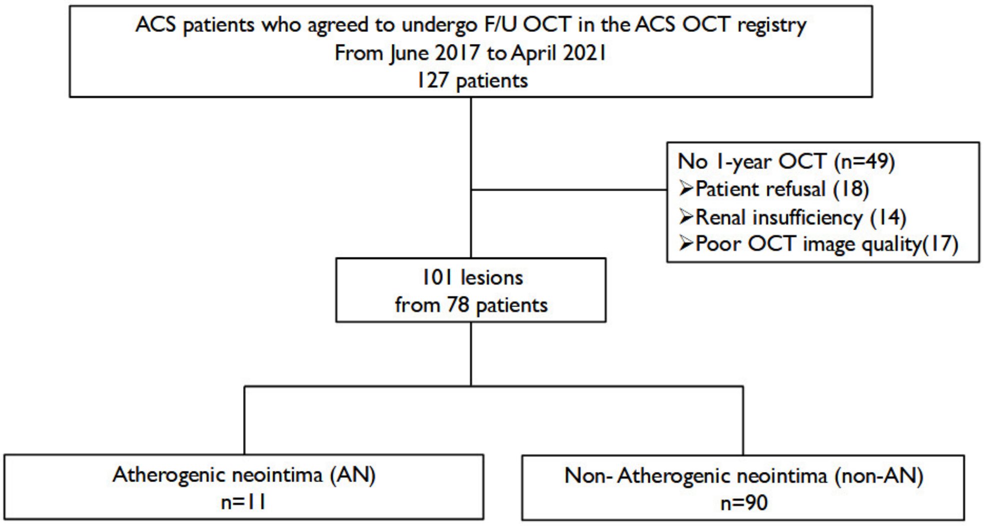

Supplemental Figure S1. Study flow chart

The single-center observational cohort study enrolled 127 patients with ACS who underwent OCT-guided PCI with currently-available DES, and a total of 101 lesions from 78 patients who underwent 1-year follow-up OCT were divided into 2 groups: AN group (n = 11) and non-AN group (n = 90).

ACS: acute coronary syndrome, DES: drug-eluting stent, IP: irregular protrusion, OCT: optical coherence tomography, PCI: percutaneous coronary intervention, AN: atherogenic neointima

Figure S-2

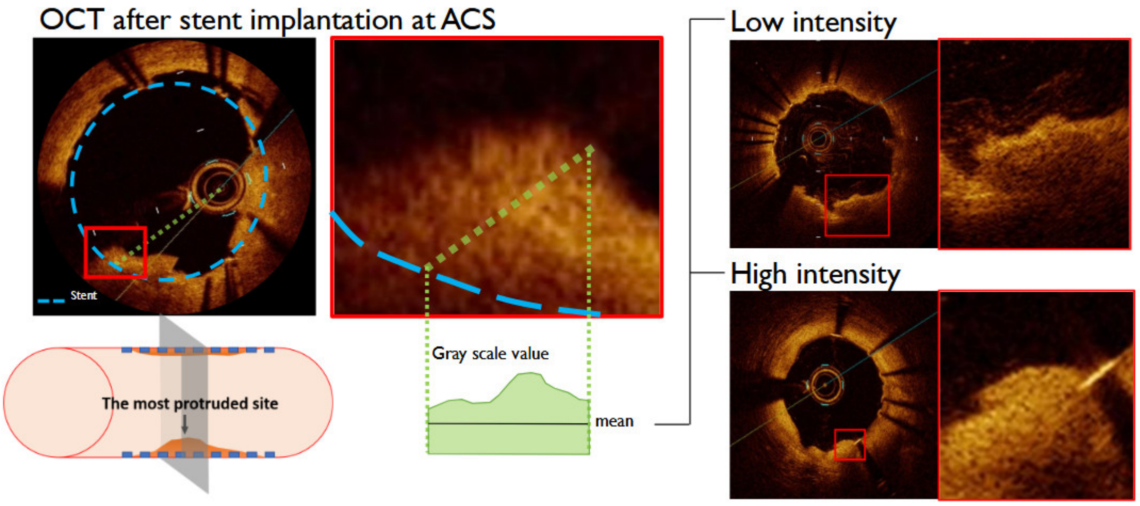

Supplemental Figure S2. Method of Gray scale value analysis

The signal intensity of the extruding tissue following stent implantation for acute coronary syndrome was measured using ImageJ. A perpendicular line was drawn from the most extruding point to the stent strut to measure the the gray scale value. The extruding plaque was classified into high intensity and low intensity based on the mean gray scale value, with the cut-off value determined using ROC curve.

OCT: optical coherence tomography; ACS: acute coronary syndrome; ROC: Receiver operating characteristic

Figure S-3

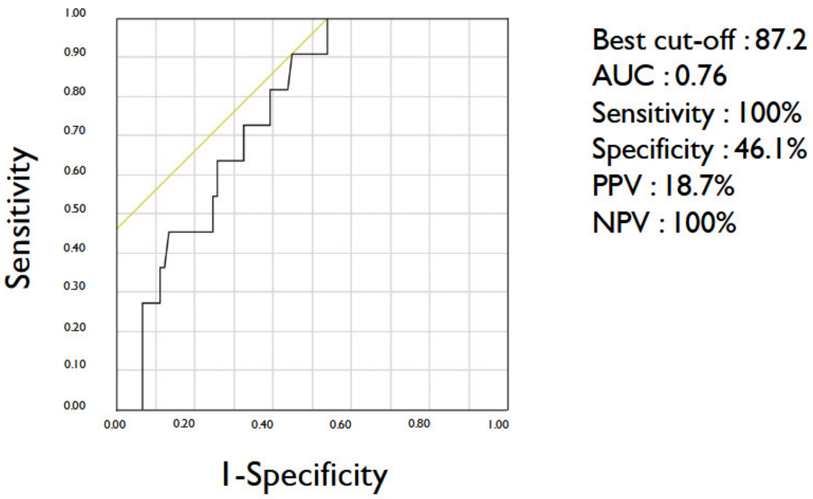

Supplemental Figure S3. Receiver operating characteristic curve of mean gray scale value

Receiver operating characteristics (ROC) curve analysis of mean gray value revealed the predictive cut-off value for the development of atherogenic neointima. The optimal cut-off value of that was 87.2. (sensitivity, 100%; specificity, 46.1%; area under the curve, 0.76; $p<0.01$)

ROC: Receiver operating characteristic

Figure S-4

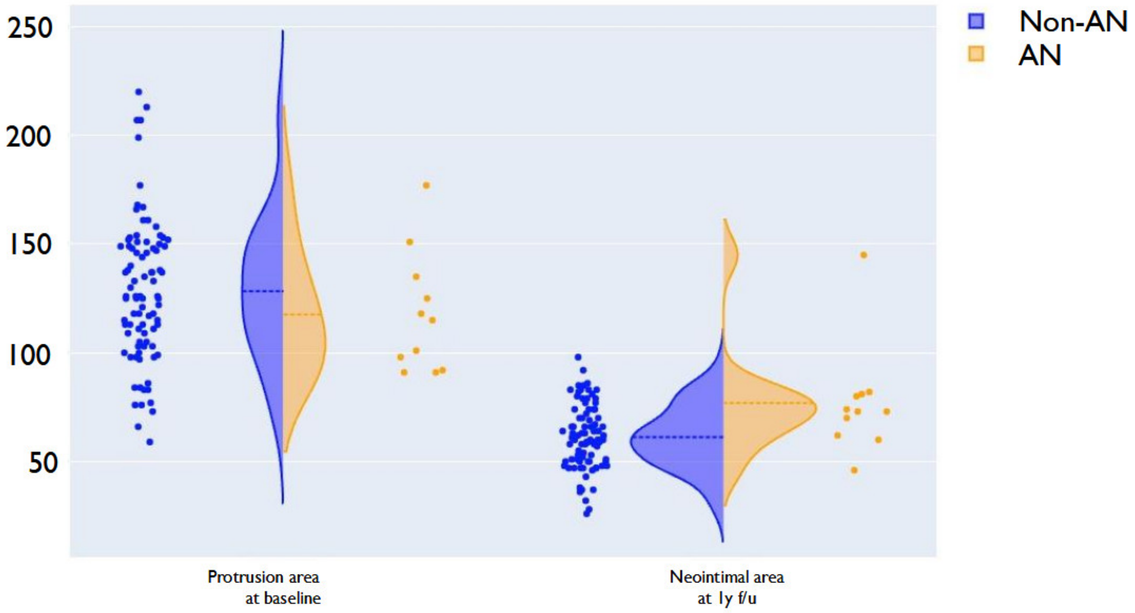

Supplemental Figure S4. Baseline and one year follow up low-density lipoprotein cholesterol between atherogenic and non-atherogenic neointima

Baseline low-density lipoprotein cholesterol levels were comparable between the atherogenic neointima (AN) and non-AN groups, however 1-year follow-up LDL levels were significantly higher in the AN group (76.9 ± 4.80 mg/dL vs. 61.2 ± 1.70 mg/dL, $p = 0.02$)

AN: atherogenic neointima; LDL: low-density lipoprotein cholesterol
